# Improved maize reference genome with single-molecule technologies

**DOI:** 10.1038/nature22971

**Published:** 2017-06-12

**Authors:** Yinping Jiao, Paul Peluso, Jinghua Shi, Tiffany Liang, Michelle C. Stitzer, Bo Wang, Michael S. Campbell, Joshua C. Stein, Xuehong Wei, Chen-Shan Chin, Katherine Guill, Michael Regulski, Sunita Kumari, Andrew Olson, Jonathan Gent, Kevin L. Schneider, Thomas K. Wolfgruber, Michael R. May, Nathan M. Springer, Eric Antoniou, W. Richard McCombie, Gernot G. Presting, Michael McMullen, Jeffrey Ross-Ibarra, R. Kelly Dawe, Alex Hastie, David R. Rank, Doreen Ware

**Affiliations:** 1 Cold Spring Harbor Laboratory, Cold Spring Harbor, New York, 11724 USA; 2 Pacific Biosciences, Menlo Park, 94025 California USA; 3 BioNano Genomics, San Diego, 92121 California USA; 4 Department of Plant Sciences and Center for Population Biology, University of California, Davis, Davis, 95616 California USA; 5 USDA-ARS, Plant Genetics Research Unit, Columbia, 65211 Missouri USA; 6 University of Georgia, Athens, 30602 Georgia USA; 7 Department of Molecular Biosciences and Bioengineering, University of Hawaii, Honolulu, 96822 Hawaii USA; 8 Department of Evolution and Ecology, University of California, Davis, 95616 California USA; 9 Department of Plant Biology, University of Minnesota, St Paul, 55108 Minnesota USA; 10 Department of Plant Sciences, Center for Population Biology, and Genome Center, University of California, Davis, 95616 California USA; 11 USDA-ARS, NEA Robert W. Holley Center for Agriculture and Health, Cornell University, Ithaca, 14853 New York USA

**Keywords:** Plant sciences, Genetics, Genome informatics

## Abstract

Complete and accurate reference genomes and annotations provide fundamental tools for characterization of genetic and functional variation^1^. These resources facilitate the determination of biological processes and support translation of research findings into improved and sustainable agricultural technologies. Many reference genomes for crop plants have been generated over the past decade, but these genomes are often fragmented and missing complex repeat regions^2^. Here we report the assembly and annotation of a reference genome of maize, a genetic and agricultural model species, using single-molecule real-time sequencing and high-resolution optical mapping. Relative to the previous reference genome^3^, our assembly features a 52-fold increase in contig length and notable improvements in the assembly of intergenic spaces and centromeres. Characterization of the repetitive portion of the genome revealed more than 130,000 intact transposable elements, allowing us to identify transposable element lineage expansions that are unique to maize. Gene annotations were updated using 111,000 full-length transcripts obtained by single-molecule real-time sequencing^4^. In addition, comparative optical mapping of two other inbred maize lines revealed a prevalence of deletions in regions of low gene density and maize lineage-specific genes.

**Supplementary information:**

The online version of this article (doi:10.1038/nature22971) contains supplementary material, which is available to authorized users.

## Abstract (web summary)

An improved reference genome for maize, using single-molecule sequencing and high-resolution optical mapping, enables characterization of structural variation and repetitive regions, and identifies lineage expansions of transposable elements that are unique to maize.

**Supplementary information:**

The online version of this article (doi:10.1038/nature22971) contains supplementary material, which is available to authorized users.

## A better map of the maize genome

The maize genome was initially reported in 2009 but with some accuracy limitations. Doreen Ware and colleagues report a new reference genome for maize using single-molecule sequencing and high-resolution optical mapping. The technique shows improvements in the gene space including resolution of gaps and misassemblies and correction of order and orientation of genes. The authors characterize structural variation and repetitive regions, and identify transposable element lineage expansions unique to maize.

**Supplementary information:**

The online version of this article (doi:10.1038/nature22971) contains supplementary material, which is available to authorized users.

## Main

Maize is the most productive and widely grown crop in the world, as well as a foundational model for genetics and genomics^5^. An accurate genome assembly for maize is crucial for all forms of basic and applied research, which will enable increases in yield to feed the growing world population. The current assembly of the maize genome, based on Sanger sequencing, was first published in 2009 (ref. 3). Although this initial reference enabled rapid progress in maize genomics^1^, the original assembly is composed of more than 100,000 small contigs, many of which are arbitrarily ordered and oriented, markedly complicating detailed analysis of individual loci^6^ and impeding investigation of intergenic regions crucial to our understanding of phenotypic variation^7,8^ and genome evolution^9,10^.

**Figure 1:**
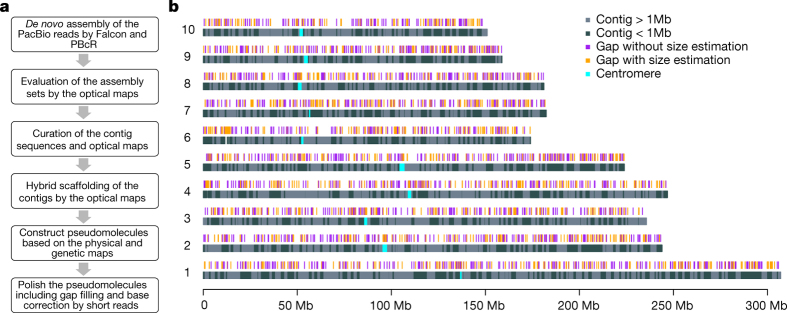
Genome assembly layout. **a**, Workflow for genome construction. **b**, Ideograms of maize B73 version 4 reference pseudomolecules. The top track shows positions of 2,522 gaps in the pseudomolecules, including 1,115 gaps in which the lengths were estimated using optical genome maps (orange), whereas the remainder (purple) have undetermined lengths. More than half of the assembly is constituted of contigs longer than 1 Mb, which are shown as light grey bars in the bottom track. PowerPoint slide Source data

**Extended Data Figure 1:**
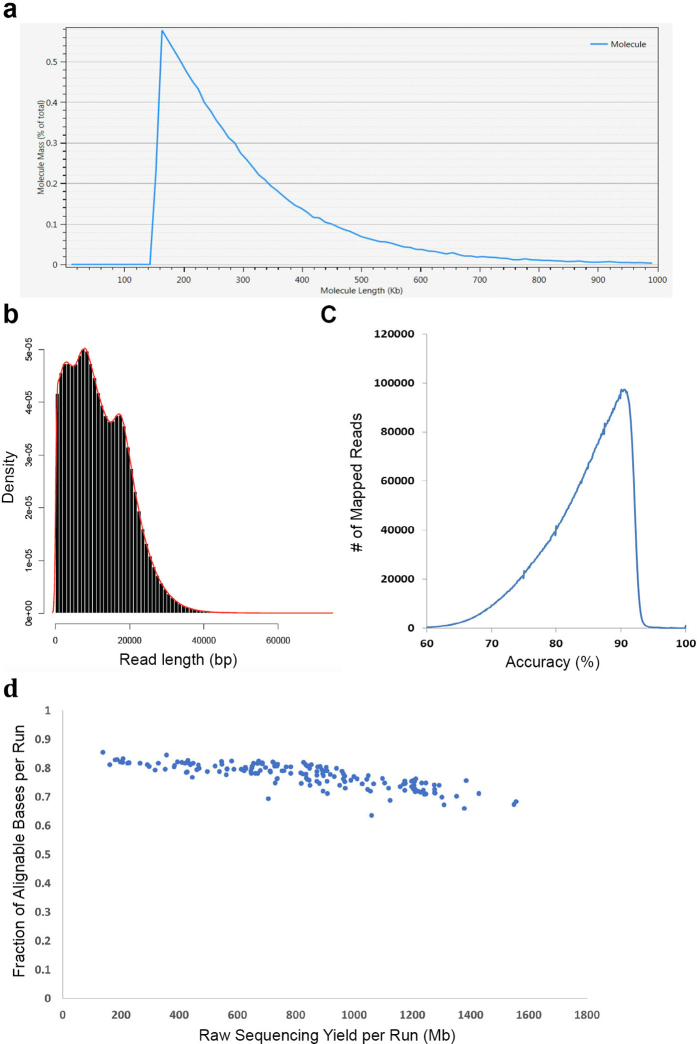
Summary of data generated for genome construction. **a**, Size distribution of single molecules for the optical maps. A total of 150 Gb (~60-fold coverage) of single-molecule raw data from BioNano chips was collected for map construction. The N50 of the single molecules was ~261 kb, and the label density was 11.6 per 100 kb. After assembly, the total size of the map reached 2.12 Gb with an N50 of 2.47 Mb. **b**, Length distribution of SMRT sequencing reads. Sequencing of 212 P6-C4 SMRT cells on the PacBio platform generated ~65-fold depth-of-coverage of the nuclear genome. Read lengths averaged 11.7 kb, with reads above 10 kb providing 53-fold depth-of-coverage. **c**, The accuracy of SMRT sequencing from a representative run. The sequencing error rate was estimated at 10% from the alignment with the maize B73 RefGen_v3 by BLASR. **d**, Plot of the fraction of alignable data per run (alignable bases/total bases per chip) versus total raw bases (per chip) for each B73 sequencing run. As the plot shows, the trend in the data suggests that as the overall per run raw base yield increases, the fraction of alignable bases decreases. This is owing to the fact that in all runs, a subset of the zero-mode waveguide (ZMWs) will initially have more than one active sequencing enzyme in the observation field at the start of the sequencing run. A ZMW with more than one active polymerase will create unalignable bases while the two polymerases are simultaneously synthesizing DNA and yield a ‘merged sequencing signal from two independent polymerases’. As the loading of a chips increases (yield of bases), the probability of having two or more polymerases in a single ZMW increases.

**Extended Data Figure 2:**
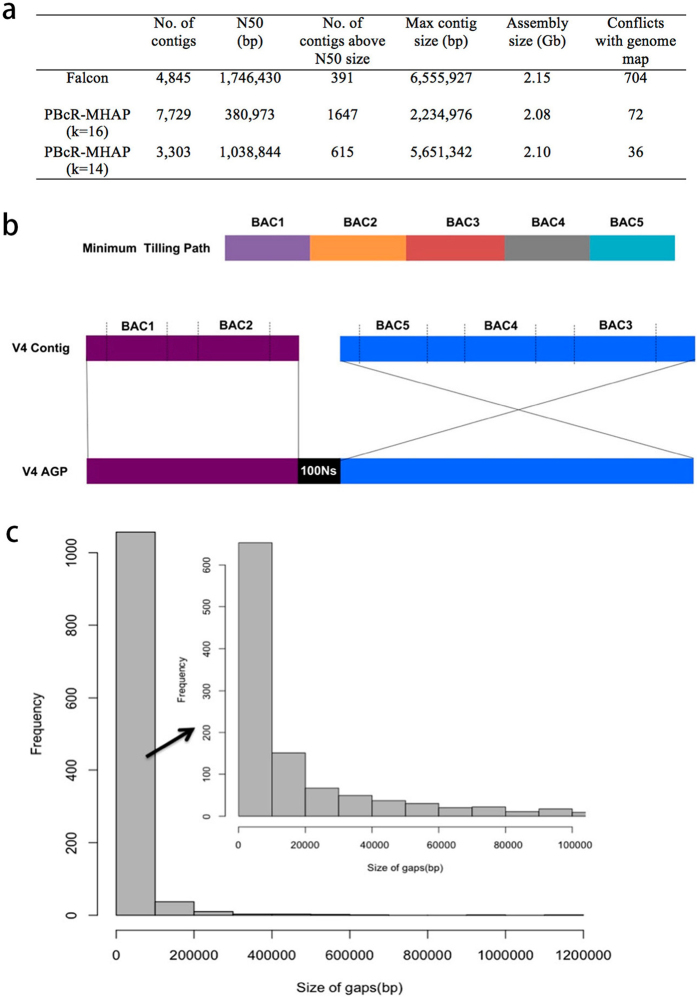
Construction of pseudomolecules. **a**, Summary of the three assembly sets. **b**, How the scaffolds were ordered according to the order of the BACs. **c**, Size distribution of gaps in the pseudomolecules estimated using the optical map.

Here we report a vastly improved *de novo* assembly and annotation of the maize reference genome (Fig. 1). On the basis of 65× single-molecule real-time sequencing (SMRT) (Extended Data Fig. 1), we assembled the genome of the maize inbred line B73 into 2,958 contigs, in which half of the total assembly is made up of contigs larger than 1.2 Mb (Table 1, Extended Data Fig. 2a). The assembly of the long reads was then integrated with a high-quality optical map (Extended Data Fig. 1, Extended Data Table 1) to create a hybrid assembly consisting of 625 scaffolds (Table 1). To build chromosome-level super-scaffolds, we combined the hybrid assembly with a minimum tiling path generated from the bacterial artificial chromosomes (BACs)^11^ and a high-density genetic map^12^ (Extended Data Fig. 2b). After gap-filling and error correction using short sequence reads, the total size of maize B73 RefGen_v4 pseudomolecules was 2,106 Mb. The new reference assembly has 2,522 gaps, of which almost half (*n* = 1,115) have optical map coverage, giving an estimated mean gap length of 27 kb (Extended Data Fig. 2c). The new maize B73 reference genome has 240-fold higher contiguity than the recently published short-read genome assembly of maize cultivar PH207 (contig N50: 1,180 kb versus 5 kb)^13^.

**Table 1:** Assembly statistics of the maize B73 RefGen_v4 genome

	Number of contigs (scaffolds)	Mean length (Mb)	N50 size (Mb)	Maximum length (Mb)	Total assembly length (Mb)
Original optical maps	1,342	1.57	2.47	12.43	2,107
Original contigs from sequence assembly	3,303	0.64	1.04	5.65	2,105
Curated optical maps	1,356	1.56	2.47	12.47	2,114
Curated contigs from sequence assembly	2,958	0.71	1.18	7.26	2,104
Optical maps in hybrid scaffolds	1,287	1.62	2.49	12.47	2,080
Contigs in hybrid scaffolds	2,696	0.77	1.19	7.26	2,075
Hybrid scaffolds	356	5.97	9.73	38.53	2,075
Hybrid scaffolds and non-scaffolded contigs	625	3.45	9.56	38.53	2,105

**Extended Data Figure 3:**
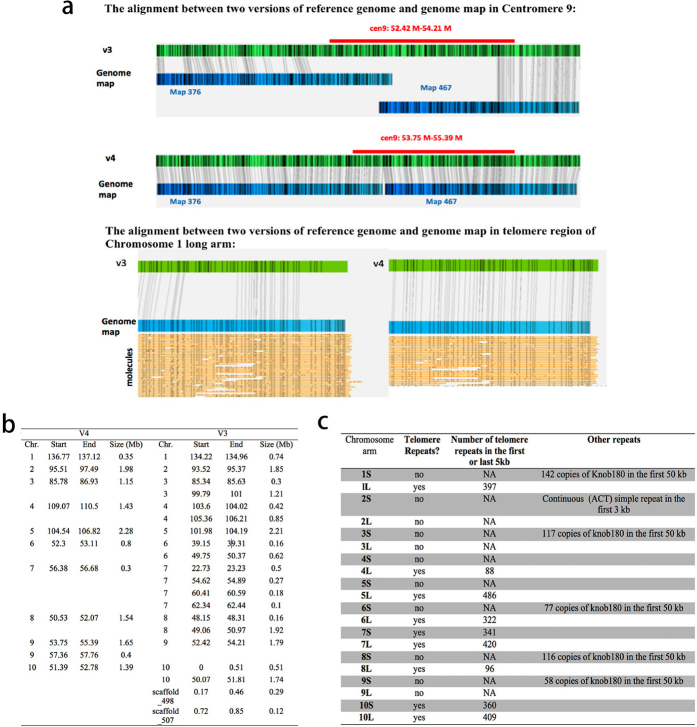
Quality assessment and comparison of the assembly in centromere and telomere regions in maize B73 RefGen_v3 and v4. **a**, Quality assessment of centromere and telomere using optical genome map. **b**, Locations of centromeres on pseudomolecules defined by ChIP–seq in the B73 RefGen_v3 and v4. **c**, Telomere repeats found in the B73 RefGen_v4 pseudomolecules.

Comparison of the new assembly to the previous BAC-based maize reference genome assembly revealed more than 99.9% sequence identity and a 52-fold increase in the mean contig length, with 84% of the BACs spanned by a single contig from the long reads assembly. Alignment of chromatin-immunoprecipitation followed by sequencing (ChIP–seq) data for the centromere-specific histone H3 (CENH3)^14^ revealed that centromeres are accurately placed and largely intact. Several previously identified^15^ megabase-sized mis-oriented pericentromeric regions were also corrected (Extended Data Fig. 3a, b). Moreover, the ends of the chromosomes are properly identified on 14 out of the 20 chromosome arms based on the presence of tandem telomeric repeats and knob 180 sequences (Extended Data Fig. 3a, c).

**Extended Data Figure 4:**
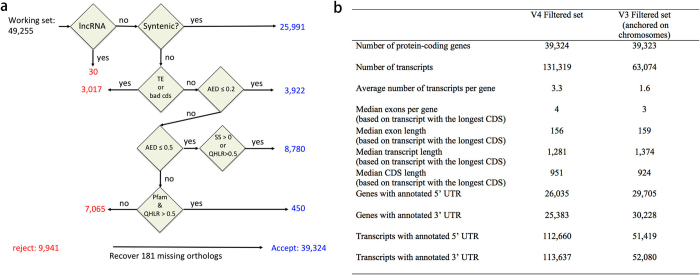
Details of the gene annotation of maize B73 RefGen_v4. **a**, The pipeline used to characterize high confidence gene models. **b**, Summary of B73 RefGen_v4 protein-coding gene annotation, and comparison with RefGen_v3 annotation.

**Extended Data Figure 5:**
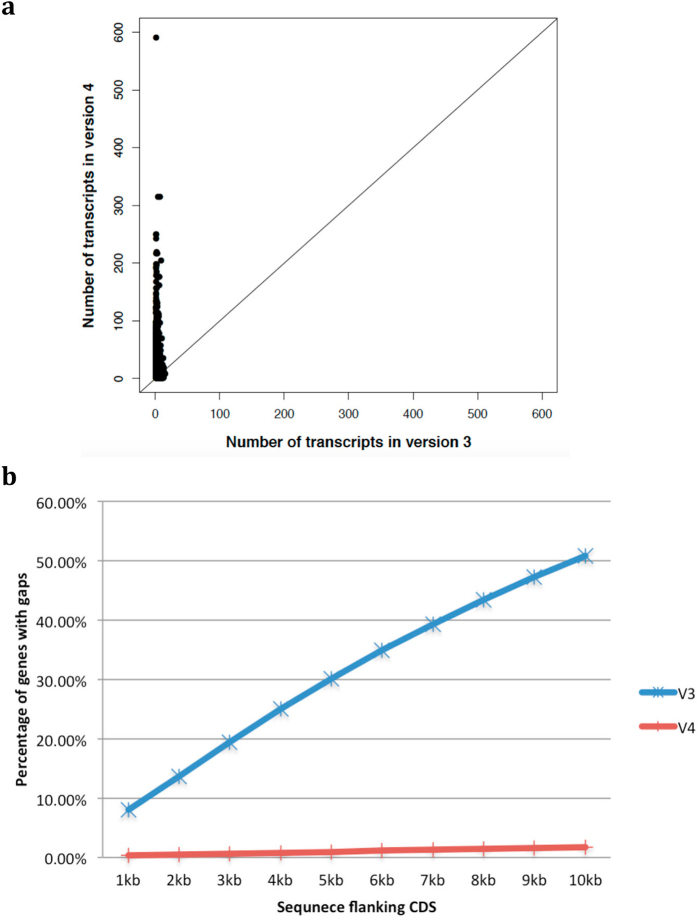
Improvement of the annotation of alternative splicing and completeness of regulatory regions of maize RefGen_v4 genes. **a**, Number of transcripts of each gene in v3 and v4 annotation. **b**, Percentages of genes with gaps in flanking regions in the v3 and v4 annotations.

Our assembly made substantial improvements in the gene space including resolution of gaps and misassemblies and correction of order and orientation of genes. We also updated the annotation of our new assembly, resulting in consolidation of gene models (Extended Data Fig. 4). Newly published full-length cDNA data^4^ improved the annotation of alternative splicing by more than doubling the number of alternative transcripts from 1.6 to 3.3 per gene (Extended Data Fig. 5a), with about 70% of genes supported by the full-length transcripts. Our reference assembly also vastly improved the coverage of regulatory sequences, decreasing the number of genes exhibiting gaps in the 3-kb region(s) flanking coding sequence from 20% to <1% (Extended Data Fig. 5b). The more complete sequence enabled notable improvements in the annotation of core promoter elements, especially the TATA-box, CCAAT-box, and Y patch motifs (Supplementary Information). Quantitative genetic analyses have shown that polymorphisms in regulatory regions explain a substantial majority of the genetic variation for many phenotypes^7,8^, suggesting that the new reference will markedly improve our ability to identify and predict functional genetic variation.

**Extended Data Figure 6:**
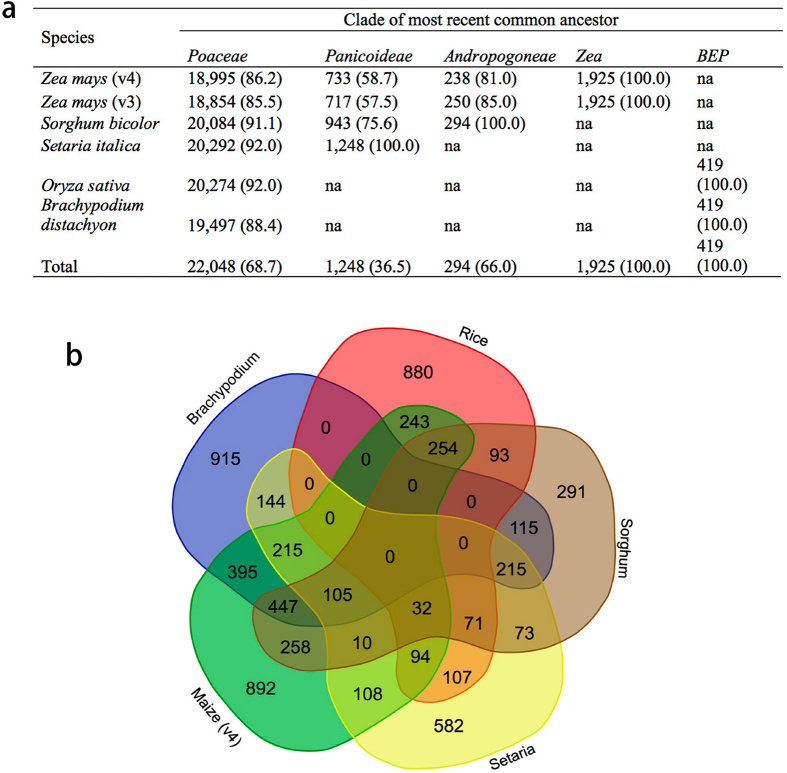
Comparative analysis of the maize B73 RefGen_v4 genes with other grasses. **a**, Species-membership in orthologue sets, giving counts and percentage of orthologue sets of which each species is a member. Numbers in parentheses give the percentage of orthologue sets with membership of all species and versions within the clade. na, not applicable. **b**, Venn diagram showing overlap of 6,539 orthologue sets rooted in the Poaceae (true grasses) that are deficient in gene membership among five species.

After its divergence from *Sorghum*, the maize lineage underwent genome doubling followed by diploidization and gene loss. Previous work showed that gene loss is biased towards one of the parental genomes^3,16^, but our new assembly and annotation instead suggest that 56% of syntenic sorghum orthologues map uniquely to the dominant maize subgenome (designated A, total size 1.16 Gb), whereas only 24% map uniquely to subgenome B (total size 0.63 Gb). Gene loss in maize has primarily been considered in the context of polyploidy and functional redundancy^16^, but we found that despite its polyploidy, maize has lost a larger proportion (14%) of the 22,048 ancestral gene orthologues than any of the other four grass species evaluated to date (*Sorghum*, rice, *Brachypodium distachyon* and *Setaria italica*; Extended Data Fig. 6). Nearly one-third of these losses are specific to maize, and analysis of a restricted high-confidence set revealed enrichment for genes involved in biotic and abiotic stresses (Extended Data Table 2), for example, NB-ARC domain disease-resistance genes^17^ and the serpin protease inhibitor involved in pathogen defence and programmed cell death^18^.

**Table 2:** Structural variations from optical maps of two maize lines

	Ki11 map versus B73 RefGen_v4	W22 maps versus B73 RefGen_v4
Total size of genome map (Mb)	2,216	2,280
Map aligned to reference genome (Mb)	722	893
Reference genome covered by map (Mb)	694	861
Region in B73 with insertion and deletion (Mb)	223	221
Ratio of region with insertion and deletion (%)	32.15	25.67
Number of insertions	1,794	1,614
Average insertion size (bp)	21,510	21,470
Number of deletions	1,701	1,597
Average deletion size (bp)	18,340	20,120
Number of deletion regions potentially affecting genes	636	621

**Figure 2:**
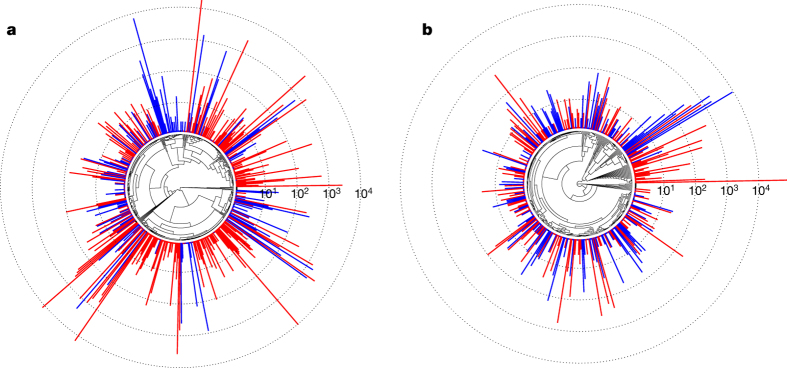
Phylogeny of maize and sorghum LTR retrotransposon families. **a**, **b**, Both Ty3/Gypsy (**a**) and Ty1/Copia (**b**) superfamilies are present at higher copy number in maize (red) than in sorghum (blue). Bars (log_10_-scaled) depict family copy numbers. PowerPoint slide Source data

Transposable elements were first reported in maize^19^ and have since been shown to have important roles in shaping genome evolution and gene regulatory networks of many species^20^. Most of the maize genome is derived from transposable elements^3,21^, and careful study of a few regions has revealed a characteristic structure of sequentially nested retrotransposons^21,22^ and the effect of deletions and recombination on retrotransposon evolution^23^. In the annotation of the original maize assembly, however, fewer than 1% of long terminal repeat (LTR) retrotransposon copies were intact^24^. By applying a new homology-independent annotation pipeline to our assembly (Extended Data Table 3), we identified 1,268 Mb (130,604 copies) of structurally intact retrotransposons, of which 661 Mb (70,035 copies) are nested retrotransposon copies disrupted by the insertion of other transposable elements, 8.7 Mb (14,041 copies) are DNA terminal inverted repeat transposons, and 76 Mb (21,095 copies) are helitrons. To understand the evolutionary history of maize LTR retrotransposons, we also applied our annotation pipeline to the sorghum reference genome, and used reverse transcriptase protein domain sequences that were accessible owing to the improved assembly of the internal protein coding domains of maize LTR retrotransposons to reconstruct the phylogeny of maize and sorghum LTR retrotransposon families. Despite a higher overall rate of diversification of LTR transposable elements in the maize lineage consistent with its larger genome size, differences in LTR retrotransposon content between genomes were primarily the result of marked expansion of distinct families in both lineages (Fig. 2).

**Figure 3:**
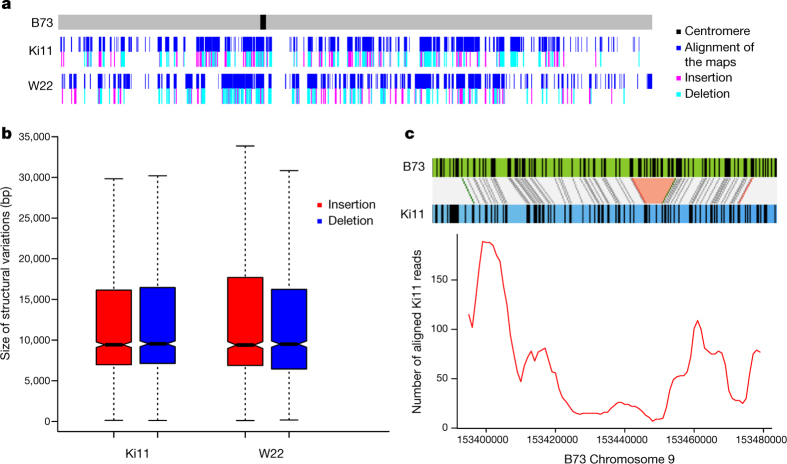
Structural variation from Ki11 and W22. **a**, Alignment and structural variation called from Ki11 and W22 optical maps on chromosome 10. **b**, Size distribution of the insertion and deletions in Ki11 and W22. **c**, Example of using short-read alignment to verify a missing region mapped in Ki11. PowerPoint slide Source data

**Extended Data Figure 7:**
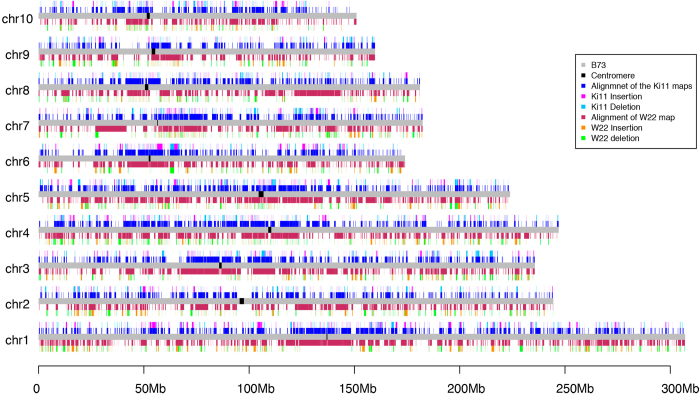
Structural variation characterized from the Ki11 and W22 optical maps.

Maize exhibits tremendous genetic diversity^25^, and both nucleotide polymorphisms and structural variations have important roles in its phenotypic variation^10,26^. However, genome-wide patterns of structural variation in plant genomes are difficult to assess^27^, and previous efforts have relied on short-read mapping, which misses the vast majority of intergenic spaces where most rearrangements are likely to occur^10^. To investigate structural variation at a genome-wide scale, we generated optical maps (Extended Data Table 1) for two additional maize inbred lines: the tropical line Ki11, one of the founders of the maize nested association mapping (NAM) population^28^, and W22, which has served as a foundation for studies of maize genetics^29^. Owing to the high degree of genomic diversity among these lines, only 32% of the assembled 2,216 Mb map of Ki11 and 39% of the 2,280 Mb W22 map could be mapped to our new B73 reference via common restriction patterns (Table 2, Fig. 3a and Extended Data Fig. 7). The high density of alignments across and near many of the exceedingly retrotransposon-rich centromeres reflects the comparatively low genetic diversity of most centromeres in domesticated maize^15^ and illustrates the ability of the combined optical mapping/single-molecule sequencing methodology to traverse large repeat-rich regions. Within the aligned regions, approximately 32% of the Ki11 and 26% of the W22 optical maps exhibited clear evidence of structural variation, including 3,408 insertions and 3,298 deletions (Table 2). The average indel size was approximately 20 kb, with a range from 1 kb to over 1 Mb (Fig. 3b). More than 90% of the indels were unique to one inbred or the other, indicating a high level of structural diversity in maize. As short-read sequence data are available from both Ki11 and W22 (ref. 10), we analysed 1,451 of the largest (>10 kb) deletions and found that 1,083 were supported by a clear reduction in read depth (Fig. 3c). The confirmed deletions occurred in regions of low gene density (4.4 genes per megabase compared to a genome-wide average of 18.7 genes per megabase). One-third (83 out of 257) of the genes missing in Ki11 or W22 lack putative orthologues in all four grasses (rice, sorghum, *Brachypodium* and *Setaria*), consistent with previous data^30^.

Although maize is often considered to be a large-genome crop, most major food crops have even larger genomes with more complex repeat landscapes^2^. Our improved assembly of the B73 genome, generated using single-molecule technologies, demonstrates that additional assemblies of other maize inbred lines and similar high-quality assemblies of other repeat-rich and large-genome plants are feasible. Further high-quality assemblies will in turn extend our understanding of the genetic diversity that forms the basis of the phenotypic diversity in maize and other economically important plants.

## Methods

No statistical methods were used to predetermine sample size. The experiments were not randomized, and investigators were not blinded to allocation during experiments and outcome assessment.

### Whole-genome sequencing using SMRT technology

DNA samples for SMRT sequencing were prepared using maize inbred line B73 from NCRPIS (PI550473), grown at University of Missouri. Seeds of this line were deposited at NCRPIS (tracking number PI677128). Etiolated seedlings were grown for 4–6 days in Pro-Mix at 37 °C in darkness to minimize chloroplast DNA. Batches of ~10 g were snap-frozen in liquid nitrogen. DNA was extracted following the PacBio protocol ‘Preparing Arabidopsis Genomic DNA for Size-Selected ~20 kb SMRTbell Libraries’ (http://www.pacb.com/wp-content/uploads/2015/09/Shared-Protocol-Preparing-Arabidopsis-DNA-for-20-kb-SMRTbell-Libraries.pdf).

Genomic DNA was sheared to a size range of 15–40 kb using either G-tubes (Covaris) or a Megarupter device (Diagenode), and enzymatically repaired and converted into SMRTbell template libraries as recommended by Pacific Biosciences. In brief, hairpin adapters were ligated, after which the remaining damaged DNA fragments and those without adapters at both ends were eliminated by digestion with exonucleases. The resulting SMRTbell templates were size-selected by Blue Pippin electrophoresis (Sage Sciences) and templates ranging from 15 to 50 kb, were sequenced on a PacBio RS II instrument using P6-C4 sequencing chemistry. To acquire long reads, all data were collected as either 5- or 6-h sequencing videos.

### Construction of optical genome maps using the Irys system

High-molecular mass genomic DNA was isolated from 3 g of young ear tissue after fixing with 2% formaldehyde. Nuclei were purified and lysed in embedded agarose as previously described^31^. DNA was labelled at Nt.BspQI sites using the IrysPrep kit. Molecules collected from BioNano chips were *de novo* assembled as previously described^32^ using ‘optArgument_human’.

### *De novo* assembly of the genome sequencing data

*De novo* assembly of the long reads from SMRT Sequencing was performed using two assemblers: the Celera Assembler PBcR –MHAP pipeline^33^ and Falcon^34^ with different parameter settings. Quiver from SMRT Analysis v2.3.0 was used to polish base calling of contigs. The three independent assemblies were evaluated by aligning with the optical genome maps.

Contamination of contigs by bacterial and plasmid genomes was eliminated using the NCBI GenBank submission system^35^. Curation of the assembly, including resolution of conflicts between the contigs and the optical map and removal of redundancy at the edges of contigs, is described in the Supplementary Information.

### Hybrid scaffold construction

To create hybrid scaffolds, curated sequence contigs and optical maps were aligned and merged with RefAligner^32^ (*P* < 1 × 10^−11^). These initial hybrid scaffolds were aligned again to the sequence contigs using a less stringent *P* value (1 × 10^−8^), and those contigs not previously merged were added if they aligned over 50% of their length and without overlapping previously merged contigs, thereby generating final hybrid scaffolds.

### Pseudomolecule construction

Sequences from BACs on the physical map that were used to build the maize v3 pseudomolecules were aligned to contigs using MUMMER package^36^ with the following parameter settings: ‘-l(minimum length of a single match) 100 -c(the minimum length of a cluster of matches) 1000’. To only use unique hits as markers, alignment hits were filtered with the following parameters: ‘-i(the minimum alignment identity) 98 -l(the minimum alignment length) 10000’. Scaffolds were then ordered and oriented into pseudochromosomes using the order of BACs as a guide. For quality control, we mapped the SNP markers from a genetic map built from an intermated maize recombinant inbred line population (Mo17 × B73)^10^. Contigs with markers not located in pseudochromosomes from the physical map were placed into the AGP (A Golden Path) using the genetic map.

### Further polishing of pseudomolecules

Raw pseudomolecules were subjected to gap filling using Pbjelly (-maxTrim = 0, -minReads = 2) and polished again using Quiver (SMRT Analysis v2.3.0). To increase the accuracy of the base calls, we performed two lanes of sequencing on the same genomic DNA sample (library size = 450 bp) using Illumina 2500 Rapid run, which generated about 100-fold 2 × 250 paired-end (PE) data. Reads were aligned to the assembly using BWA-mem^37^. Sequence error correction was performed with the Pilon pipeline^38^, after aligning reads with BWA-mem^37^ and parsing with SAMtools^39^, using sequence and alignment quality scores above 20.

### Annotation

For comprehensive annotation of transposable elements, we designed a structural identification pipeline incorporating several tools, including LTRharvest^40^, LTRdigest^41^, SINE-Finder^42^, MGEScan-non-LTR^43^, MITE-hunter^44^, HelitronScanner^45^, and others (details in Supplementary Information). The scripts, parameters, and intermediate files of each transposable element superfamily are available at https://github.com/mcstitzer/maize_v4_TE_annotation.

The MAKER-P pipeline was used to annotate protein-coding genes^46^, integrating *ab initio* prediction with publicly available evidence from full-length cDNA^47^, *de novo* assembled transcripts from short-read mRNA sequencing (mRNA-seq)^48^, isoform-sequencing (Iso-Seq) full-length transcripts^14^, and proteins from other species. The gene models were filtered to remove transposons and low-confidence predictions. Additional alternative transcript isoforms were obtained from the Iso-Seq data. Further details on annotations, core promoter analysis, and comparative phylogenomics are described in Supplementary Information.

### Structural variation

Leaves were used to prepare high molecular mass DNA and optical genome maps were constructed as described above for B73. Structural variant calls were generated based on alignment to the reference map B73 v4 chromosomal assembly using the multiple local alignment algorithm (RefSplit)^32^. A structural variant was identified as an alignment outlier^32,49^, defined as two well-aligned regions separated by a poorly aligned region with a large size difference between the reference genome and the map or by one or more unaligned sites, or alternatively as a gap between two local alignments. A confidence score was generated by comparing the non-normalized *P* values of the two well-aligned regions and the non-normalized log-likelihood ratio^50^ of the unaligned or poorly aligned region. With a confidence score threshold of 3, RefSplit is sensitive to insertions and deletions as small as 100 bp (events smaller than 1 kb are generally compound or substitution and include label changes, not just spacing differences) and other changes such as inversions and complex events which could be balanced. Insertion and deletion calls were based on an alignment outlier *P*-value threshold of 1 × 10^−4^. Insertions or deletions that crossed gaps in the B73 pseudomolecules, or that were heterozygous in the optical genome maps, were excluded. Considering the resolution of the BioNano optical map, only insertion and deletions larger than 100 bp were used for subsequent analyses. To obtain high-confidence deletion sequences, sequencing reads from the maize HapMap2 project^8^ for Ki11 and W22 were aligned to our new B73 v4 reference genome using Bowtie2 (ref. 51). Read depth (minimum mapping quality >20) was calculated in 10-kb windows with step size of 1 kb. Windows with read depth below 10 in Ki11 and 20 in W22 (sequencing depths for Ki11 and W22 were 2.32× and 4.04×, respectively) in the deleted region were retained for further analysis.

### Data availability

Raw reads, genome assembly sequences, and gene annotations have been deposited at the NCBI under BioProject number PRJNA10769 and BioSample number SAMN04296295. PacBio whole-genome sequencing data and Illumina data were deposited in the NCBI SRA database under accessions SRX1472849 and SRX1452310, respectively. The GenBank accession number of the genome assembly and annotation is LPUQ00000000. A genome browser including genome feature tracks and ftp is available from Gramene: http://ensembl.gramene.org/Zea_mays/Info/Index. All other data are available from the corresponding author upon reasonable request.

## Supplementary information

Supplementary Information This file contains the Supplementary Methods and references. (PDF 770 kb)

## Figures and Tables

**Extended Data Table 1:**
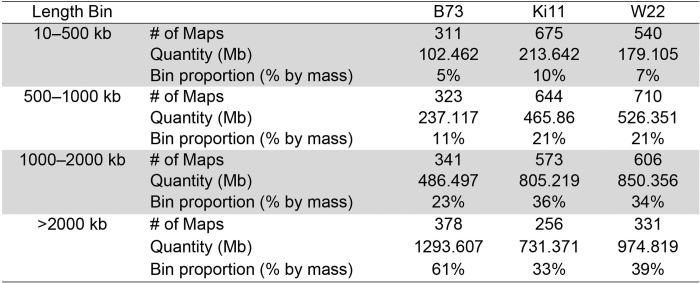
Summary of the optical maps of three maize lines

**Extended Data Table 2:**
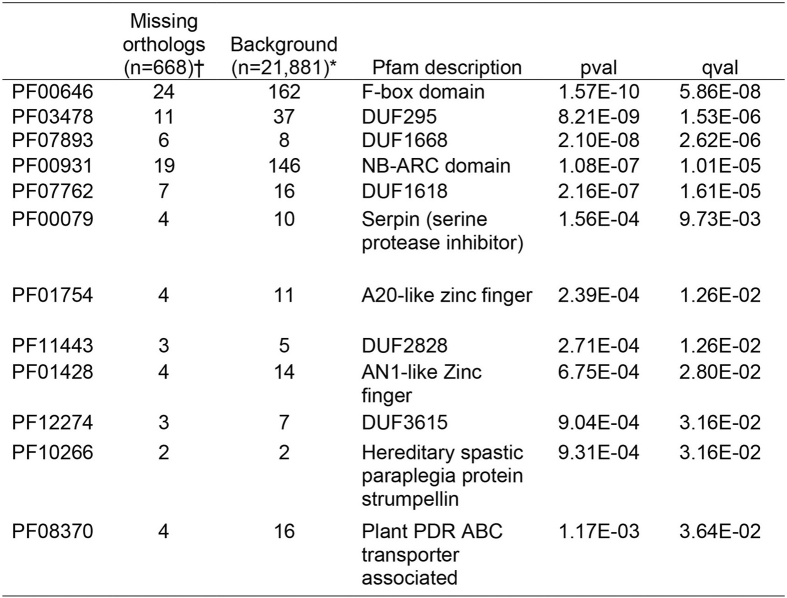
Overrepresented protein domains in sorghum genes that lack orthologues in maize but are conserved in syntenic positions in other grasses

*High-confidence sorghum genes with syntenic orthologues in rice, *Brachypodium* or *Setaria* outgroup species.

†Subset of background with no annotated orthologues in either maize v3 or v4 reference assemblies, have <50% LASTZ alignment coverage with v4, and fall within synteny blocks that map to singular assembly contigs in both the A and B subgenomes of maize. Only significantly enriched cases are shown, based on hypergeometric distribution followed by FDR correction.

**Extended Data Table 3:**
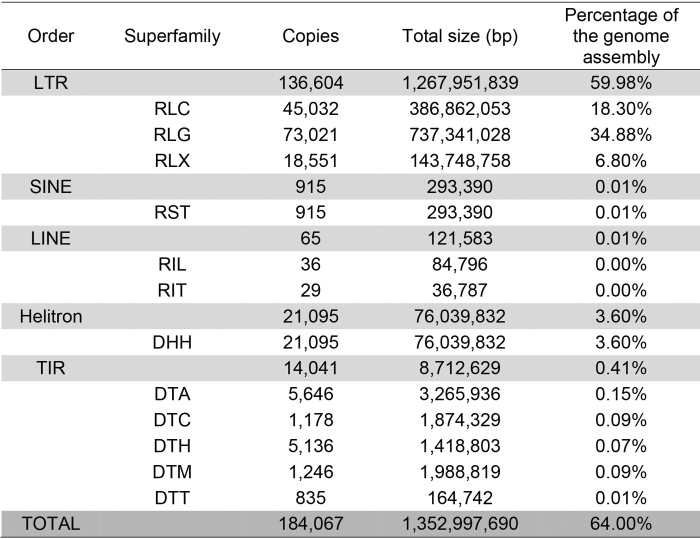
Structural annotation of transposable elements

## Data Availability

BioProject
PRJNA10769 NCBI Reference Sequence
LPUQ00000000
Sequence Read Archive
SRX1452310
SRX1472849

## PowerPoint slides

PowerPoint slide for Fig. 1
PowerPoint slide for Fig. 2
PowerPoint slide for Fig. 3

## Source data

Source data to Fig. 1
Source data to Fig. 2
Source data to Fig. 3
