# Supplementary material for: Improved maize reference genome with single-molecule technologies
Source: Nature. 2017 Jun 12;546(7659):524–7. doi: 10.1038/nature22971 (PMC7052699; doi:10.1038/nature22971)
Supplement: Supplementary Information — This file contains the Supplementary Methods and references. (PDF 770 kb) [file 41586_2017_BFnature22971_MOESM1_ESM.pdf]

doi:10.1038/nature22971

Improved maize reference genome with single molecule technologies

1. Genome assembly

***De novo* assembly of the long reads**: Two assembly tools, PBcR-MHAP and FALCON, were independently evaluated for *de novo* assembly of PacBio SMRT Sequencing reads. For PBcR, following the recommended parameters for large genome assembly[1], *k*-mer lengths of 16 and 14 were used to test the performance of assembler. The assembly redundancy in the unitigs were filtered according to sequencing coverage, according to the following criteria: coverage ≥ 2 reads, and a single read must not cross more 50% of a unitig. FALCON v.0.4 (https://github.com/PacificBiosciences/FALCON-integrate/tree/0.4.0) was also used for *de novo* assembly. The overall design of FALCON follows the hierarchical genome-assembly process[2]. Instead of BLASR, daligner was used to overlap reads. To lay out contigs from the assembly graph, the error-correction module was updated, and the Celera Assembler was replaced by a string graph–based module. Due to the highly repetitive nature of the maize genome, we adopted more aggressive parameters to reduce computation time. For the full data set, only reads longer than 12 kb were corrected. To identify overlaps between raw sequences, we used "-M24 -l4800 -k18 -h480 -w8" for Daligner. Using these parameters, only overlaps longer than 4,800 bp were considered for error correction with seed matches > 480 bp.

To ascertain the quality of the three independent assemblies (FALCON, PBcR with k=16 and 14), the BioNano scaffolding pipeline NGM Hybrid Scaffold (NGM-HS) (version 4304) was used to generate an *in silico* map for sequence contigs from each assembly. The maps were aligned against the genome assemblies using RefAligner[3,4] to identify and resolve potential conflicts in sequence contigs or optical genome maps. The result showed that the PBcR–MHAP assembly (k=14) had the fewest conflicts (Extended Data Figure 2a); consequently, it was adopted as the new B73 genome reference.

Curation of the assembly: Comparison between the contigs and optical map identified 36 conflicts. Next Generation Mapping (NGM-HS) from BioNano Genomics' Irys® System was used to resolve conflicts between the sequence and optical map assemblies by cutting either assembly (option: –N 2 –B 2); cut decisions were based on chimeric scores of labels near the conflict junctions on the optical genome map. The chimeric score of a label represents the percentage of BioNano molecules that can fully align to the optical map 55 kb to the left or right of that label. If the chimeric scores of all labels within 10 kb of the conflict junction were ≥ 35, the scaffolding pipeline suggested a cut in the sequence contig. If any label in the region had a chimeric score < 35, a cut was suggested in the BioNano optical map. All proposed cuts were manually evaluated using BioNano molecule-to-genome map alignments, molecule-to-sequence contig alignments, and the BAC-based fingerprint map. Of these 36 conflicts, 18 were chimeric in the long reads assembly, and 13 were chimeric in the optical map; five were left unresolved.

Using alignments of the optical genome map, a total of 1,369 overlaps were detected among the tails of the contigs. There are two possible reasons for this: the overlaps could be repeat boundaries between contigs from the Celera assembler[5], or alternatively, nearly identical repeats could be over-collapsed in the optical map. The redundancy at the edges of nearby contigs generated by the Celera assembler was resolved as follows: if two contigs were detected to have overlap from 0.5-10 kb (based on the size of PacBio reads) by optical genome map and had sequence identity over 95% in the overlapped region, they were merged by Mininus2[6]. A total of 670 contigs were merged into 310 larger contigs.

Pseudomolecule construction: The curated 2,958 contigs were scaffolded into 625 large hybrid scaffolds and 269 contigs that are relatively small were not covered by the optical maps. Using unique BAC sequences as markers, we could order and orient 315 hybrid-scaffolds and 25 non-scaffolded contigs. In addition, we also incorporated a genetic map built from an intermated maize recombinant inbred line population (Mo17 × B73)[7] to complement pseudomolecule construction and validation. In this new AGP (A Golden Path) of the maize reference genome, a total of 331 hybrid scaffolds and 45 non-scaffolded contigs were ordered and oriented. Of 1,907 markers on the genetic map,

1,868 could be mapped to the new pseudomolecules, with only one disagreement, demonstrating the high accuracy of the AGP. During the following gap-filling procedure, 170 gaps were filled by SMRT long reads.

To ensure base-pairing accuracy and further polish the pseudomolecules, we generated ~2300Gb Illumina pair-end reads. To increase the size of reads, the paired-end library was constructed to be overlapping (~450bp library size, read length: 250bp). After merging the two reads in a pair, the average size of Illumina reads reached 400bp.These longer reads also decreased the difficulty in the alignment. About 89.7% of the assembly had good coverage for the correction (mapping and sequencing quality >20, read depth >=5). A total of 80k bases, including SNP and small Indel, were corrected, of which 91% were small indels.

Centromere identification by ChIP-seq: Peaks of CENH3 enrichment were defined by CENH3 ChIP-seq as described previously[8] using the HOMER findPeaks software[9]. Input reads from the CENH3 ChIP sample were used as controls. All reads were mapped to the genome using BWA-MEM[10]. As a first step, all reads, including potential repetitively mapping reads, were used to identify a set of putative CENH3-enriched regions; the parameters of HOMER findPeaks were set as follows: -region -size 5000 –minDist 50000 -F 8 -L 0 -C -0. A set of high-confidence peaks was then independently identified using only uniquely mapping reads (as defined by MAPQ values $\geq$ 20) with the following parameters: -region -size 5000 -F 16 -L 0 -C -0. Putative CENH3-enriched regions that were either shorter than 100 kb, or that did not overlap with at least one high-confidence peak, were discarded. To generate the final set of centromeric loci, the remaining putative CENH3-enriched regions were merged if they were less than 500 kb apart.

2. Comparison of genome assembly quality in Maize B73 RefGen_v3 and v4

The Maize Genome Sequencing Pilot Project randomly selected 100 BAC clones for high-quality sequencing, resulting in 98 curated BACs of finished quality[11]. These BACs were used for the detailed evaluation of the assembly quality of maize v4 genome. In total, 25 of the 28 fully completed BACs were spanned by a single contig in RefGen_v4, with identity above 99.9%. In addition, the maize pilot sequence contains 57 BACs with

ordered contigs and gaps. The gaps of 46 BACs could be closed by a single contig in RefGen_v4.

Several gene models with assembly errors in the maize B73 RefGen_v3 have been corrected in the current maize genome. For example, the *rgh3* locus (JN692485.1) was involved in an assembly error that arose due to incorrect ordering and orientation of contigs in the BAC sequence, resulting in mis-annotation of this gene as two distinct gene models[12]. This problem was successfully fixed in the v4 assembly. Due to correction of such errors and the increase in contiguity described above, the RefGen_v4 assembly is much more robust as a reference genome than the old BAC sequences.

3. Gene annotation

Generation of a working gene set: MAKER-P version 3.1[13] was used to annotate the maize RefGen_v4 genome. As evidence, we used all annotated proteins from *Sorghum bicolor*, *Oryza sativa*, *Setaria italica*, *Brachypodium distachyon*, and *Arabidopsis thaliana*, downloaded from Gramene.org release 48[14]. For transcript evidence, the 111,151 high quality transcripts from Iso-seq were further polished by illumina RNA-seq reads generated from same tissues[15] using Ectools (https://github.com/jgurtowski/ectools). Another set of 69,163 publicly available full-length cDNAs deposited in Genbank[16], a total of 1,574,442 Trinity-assembled transcripts from 94 B73 RNA-Seq experiments[17], and 112,963 transcripts assembled from deep sequencing of a B73 seedling[18] were also included as transcript evidence. For gene prediction, we used Augustus[19] and FGENESH (http://www.softberry.com/berry.phtml) trained on maize and monocots, respectively. For repeat masking, we used RepeatMasker and the B73-specific TE exemplars[20]. Helitron elements and captured exons within pack-MULES were removed from this library to prevent the masking of non–TE-related protein-coding genes. Additional masking was performed using a set of known TE-derived proteins distributed with the MAKER software package[13].

The final annotation set was built iteratively. The first step, which included all of the protein evidence, the full-length cDNAs from GenBank, and the Iso-Seq data, generated 34,088 genes with 56,671 transcripts. For the second step, the gene models from the first pass were given back to MAKER as models, allowing them to persist

unchanged in the annotation set. Next, the additional transcript evidence derived from short reads was included. This step generated an additional 9,548 genes with 11,475 transcripts. To retain as many genes as possible from the v3 annotations, the third pass added the previously annotated B73 transcripts and protein translations from the v3 assembly as evidence. This step added 5,449 genes with 5,947 transcripts. MAKER-P is conservative in annotating alternate transcripts. Additionally, transcripts that contain large intron retentions, non-canonical splicing, or are expressed at low levels are also difficult to annotate confidently by computational methods. However, the single-molecule Iso-Seq transcript sequencing method can unambiguously identify these hard-to-annotate transcripts. By including the additional unique Iso-Seq transcripts into the gene models from step 3, we generated a protein-coding gene annotation set of 49,085 genes and 161,680 transcripts (referred to as the working set).

Compara gene tree construction: The Ensembl Compara gene tree pipeline[21] was used to define gene families, construct phylogenetic gene trees, and infer orthologs and paralogs. Updated protocols used in the Ensembl version 81 software are detailed elsewhere (http://jul2015.archive.ensembl.org/info/genome/compara/homology_method.html). The analysis included annotated protein-coding genes from both the v3 and v4 gene sets of maize B73, as well as 17 additional species (five monocots, four dicots, one basal angiosperm, three lower plants, and four non-plants), which were downloaded from the Ensembl core databases within Gramene Release-41. Tree reconciliation to classify duplication and speciation nodes, and the assignment of taxon levels to nodes, used the following input species tree derived from the NCBI Taxonomy database[21]: ((((((((((sorghum_bicolor,(zea_mays_v3,zea_mays_v4)N)Andropogoneae,setaria_italica)Panicoideae,(brachypodium_distachyon,oryza_sativa)BEP_clade)Poaceae,musa_acuminata)commelinids,((((arabidopsis_thaliana,glycine_max),vitis_vinifera))rosids,solanum_lycopersicum)Eudicot)Mesangiospermae,amborella_trichopoda)Magnoliophyta,selaginella_moellendorffii)Tracheophyta,physcomitrella_patens)Embryophyta,chlamydomonas_reinhardtii)Viridiplantae,(((caenorhabditis_elegans,drosophila_melanogaster)Ecdysozoa,homo_sapiens)Bilateria,saccharomyces_cerevisiae)Opisthokonta)Eukaryota;.

Synteny maps, which relate collinear chains of orthologous genes between two genomes, were built using DAGchainer[22] in combination with other previously described methods[20,23].

Generation of the filtered gene set: The working set of protein-coding gene annotations is expected to contain TEs that were not masked prior to annotation, long noncoding RNAs annotated as protein-coding genes, and annotations with little supporting evidence. We filtered the working set based on evidentiary support, transposon screening, long non-coding RNA screening, homology support, and valid CDSs. The approach is schematically represented in Extended data Figure 4a.

tRNA annotation: tRNAs were identified using tRNAscan-SE[24] within the MAKER-P framework[25]. A total of 2,305 tRNAs were identified: 1,451 decode standard amino acids, four decode seleno-Cys, seven are putative suppressors, 13 contain an undeterminable anti-codon sequence, and 830 are apparent pseudogenes. Compared to the v3 assembly, v4 contains 59 additional complete tRNAs and 54 additional putative tRNA pseudogenes. This increase in identifiable tRNAs provides further evidence that v4 is a more complete genome assembly than v3.

4. Comparison of gene annotation between RefGen_v3 and v4

Alignment of v3 genes to the v4 genome: We used two pipelines to map the v3 genes to the v4 genome, Genome Assembly Converter and Mummer pipeline[26]. In Genome Assembly Converter, the ATAC pipeline[27] was used to create the alignment chain file between two assemblies, and then CrossMap[28] was used to convert the coordinates of the v3 gene annotation. Due to the complexity of repeats in maize genome, only the one-to-one alignment blocks were saved to build the chain. In the chain file, the v3 genome covered 89.7% of v4 genome, whereas v4 covered 92.5% of v3 genome. A chromosome-to-chromosome alignment was first performed using Mummer to map the v3 genes to the v4 genome. Genes from v3 that could not be mapped to the same chromosome in v4 were then aligned to the whole v4 assembly. Only unique hits with identity above 98% and 100% coverage were retained for merging with the Genome Assembly Converter pipeline. Disagreements between the two pipelines were resolved as follows: if the

Genome Assembly Converter pipeline had 100% coverage for a given gene, then those coordinates were kept; otherwise, the result from the Mummer alignment was used.

Alignment of the RefGen_v3 and v4 genome assemblies indicated that the two versions are highly consistent with each other in gene space. A total of 36,725 (94%) v3 gene models could be mapped to the new RefGen_v4 genome without sequence changes. Most of the remaining v3 genes (1,356) could be mapped, but crossed multiple contigs in RefGen_v3, with gaps; consequently, it is very likely that they were incorrectly assembled in v3. In RefGen_v4, most of these genes were contained within continuous sequences, indicating the improvement of the genomic sequences of these genes. In addition, 92 of the 146 genes previously unanchored in RefGen_v3 were anchored to chromosomes in the RefGen_v4 assembly.

Core promoter elements: Core promoter elements were analyzed in both RefGen_v3 and v4 with a published pipeline[29,30]. Comparison of core promoter elements, especially the TATA-box, CCAAT-box, and Y patch in the new assembly to those in the previously published assembly revealed 17.5% of genes in the new assembly contained a TATA-box, whereas in the previous assembly only 12.8% genes contained this element. Similarly, 7.2% genes contained a CCAAT-box and 58.17% contained Y patch in maize B73 RefGen_v4, versus 2.4% and 41.5%, respectively in v3.

Gene orientation: Of 30,926 genes that could be mapped between the v3 and v4 annotations, 2,151 genes were switched to a different strand. To evaluate this, we compared gene orientation to sorghum orthologs within syntenic blocks. Among 652 genes that could be tracked in this manner, the orientation of 589 (90.3%) was conserved with sorghum. Thus, in the vast majority of cases, the re-orientation of a gene in v4 brought the configuration into closer agreement with sorghum, further lending confidence to strand reassignments of v4 genes.

Identification of missing genes in maize genome: We identified 22,048 orthologous gene sets that originated prior to, or within, the grass common ancestor, and cataloged deficiencies in gene content among annotations of the five grass species (maize, rice, sorghum, *Setaria*, and *Brachypodium*). Of these sets, ~69% were found in all five species, and of individual species, rice, *Setaria*, and sorghum had the most complete representation, possessing from 91% to 92% of ortholog sets. By contrast, despite the

fact that maize is a product of whole-genome duplication, maize genes were found in only 86% of ortholog sets, representing a deficit of over 3,000 genes. To minimize artifacts, we restricted analysis to 592 ortholog sets containing 668 sorghum genes that 1) are syntenic with an outgroup species (either rice, *Brachypodium*, or *Setaria*), 2) are flanked by genes contained within a synteny block that maps to a single maize contig in both the A and B subgenomes, and 3) lack alignment of CDS features to the v4 reference.

5. Structural identification of transposable elements

LTR retrotransposons: LTR retrotransposons were identified using LTRharvest[31] and LTRdigest[32]. LTRharvest searches sequence data for structural characteristics of LTR retrotransposons; in an analysis of the *Drosophila* X chromosome, it was shown to be the most sensitive among available structural search tools[33]. To be consistent with known LTR retrotransposons in maize, we adjusted default parameters including LTR length (100–7000 bp) and element length (1000–20000 bp). All searches required target site duplications (TSDs) of 4–6 bp (allowing one mismatch) and a 2-nt inverted motif at the terminal ends of each LTR (5' TG..CA 3', allowing one mismatch). If multiple overlapping elements were found, the one with the highest percent identity between LTRs was chosen with the '-overlaps best' option.

The resultant TE models were further annotated with LTRdigest[32], which identifies sequence features such as primer binding site, polypurine tract, and protein domains associated with previously identified retrotransposons from any organism. We used all eukaryotic tRNA entries from the UCSC gtRNA database to predict primer binding sites, and amino-acid HMM profiles of retrotransposon-associated proteins as deposited in GyDB (http://gydb.org)[34]. If RNase H, reverse transcriptase, and integrase domains were present, gene order was used to classify elements into the Ty1/Copia (integrase upstream of RNase H) and Ty3/gypsy (RNase H upstream of integrase) superfamilies.

LTR retrotransposons dominate the intergenic space of the maize genome. To capture the nested structure of these elements, generated when a newly arriving TE inserts into a TE already present at that genomic location, we computationally excised each LTR retrotransposon copy and repeated the structural search on this subtracted

pseudo-genome. We repeated this computational subtraction for 80 rounds, increasing the element length by 1000 bp for each round to accommodate sequence contributed by TE fragments and TEs of other orders.

SINE and LINE: Because SINEs are transcribed by RNA polymerase III, they are often derived from one of three classes of Pol III–transcribed molecules (tRNA, 7SL, 5s rRNA). Animal SINEs of all three classes are known, whereas plant SINEs are exclusively tRNA-derived[35]. We used SINE-finder[35] to search for tRNA-derived SINEs containing RNA polymerase III A and B boxes near the polyA tail. The default A and B box consensuses (RVTGG; GTTCRA), a 25–50 bp spacer between the A and B boxes, and a spacer of 20–500 bp between the B box and polyA tail were applied. Structural SINEs were predicted only on the forward strand of the genome. LINEs were identified using TARGeT and mTEA as below for TIR elements, using LINE exemplars and 15 bp target site duplications.

TIR: Exemplar elements from the maize TE consortium (MTEC) annotation[20] were used as nucleotide queries in TARGeT[36], a pipeline designed to recover high-copy transposon and gene families. The number of elements clustered in the PHI step was increased to 10000 copies, and 200 bp of flanking sequence on either edge of genomic matches was extracted (-p_f 200). This approach recovered candidate TE sequences, but the TE boundaries and flanking sequence were unknown. To identify the boundaries of each element, we scanned each candidate and verified the presence of terminal inverted repeat (TIR) and TSD sequences indicative of the TE superfamily (see the table below), using mTEA (https://github.com/hyphaltip/mTEA/blob/master/scripts/id_TIR_in_FASTA.pl; modified to use mafft for alignment), Although TSDs and TIRs should be identical for most superfamilies upon insertion into the genome, mutations arising at the background genomic mutation rate can generate differences. Thus, we allowed mismatches of 80% of the length of a TSD or TIR to accommodate identification of these older, degraded copies.

DNA TIR TE Superfamily TSD & TIR Classification

Superfamily	TSD Length (sequence restrictions, if any)	TIR Length (sequence restrictions, if any)
DTT *Tc1/Mariner*	2 bp (TA)	13 bp
DTA *hAT*	8 bp	11 bp
DTM *Mutator*	9 bp	40 bp
DTH *Pif/Harbinger*	3 bp (TNN)	14 bp
DTC *CACTA*	3 bp	13 bp (CACTNNNNNNNNN)

In addition, MiteHunter[37] and detectMITE[38] were used to identify *de novo* structural MITEs, searching for TIR and TSDs in genomic sequences. We filtered MITE output by TSD and TIR length, and all exemplars with TIRs and TSDs of anticipated length for the superfamily were used to search using mTEA, as described above.

Helitron: HelitronScanner[39] with default parameters was deployed to identify upstream and downstream termini of helitrons, and to join upstream and downstream termini within 200–20,000 bp of each other into helitron TE copies. We predicted helitrons in both the direct and reverse complement orientations.

Family clustering: Families were identified within each superfamily of TIR TE and order of retrotransposon using the 80–80–80 rule[40], which requires that elements within a family must share 80% homology over at least 80 base pairs of 80% of the element's functional or internal domains. For LTR retrotransposons, the 5' LTR was used to cluster families, consistent with previous annotations in maize[41]. The entire element sequence was used to group TIRs, LINEs, and SINEs, because functional domains are short, and because a large proportion of non-autonomous elements lack protein-coding domains. Because the internal regions of maize helitrons are diverse and clustering methods applied to the entire element yield almost exclusively singletons[42], we used a family classification previously applied to maize helitrons that relies on 80% identity of the 30 bp at the 3’ end of each copy[43], a region of hairpin-forming sequence important for rolling circle replication. All family definitions are consistent with those used previously in the maize genome sequencing project[20,41], although we implemented clustering of families in SiLiX[44]. Additionally, for each structurally defined TE in the genome, we assigned a unique identifier that indicates its superfamily and family.

Calculating genomic composition and resolving TE overlaps: As structural searches were run independently for each TE order, we filtered overlapping insertions in order to count each genomic position as derived from only one transposable element and generate a filtered set of TE annotations. As subsequent transposition into existing TEs causes them to occupy larger ranges along the genome, larger TEs are expected to be older. Since the chance of false homology increases as requirements of sequence identity are reduced, we filtered out LTR retrotransposons that occupy over 100kb along the genome, as these old large elements are more likely to be false positives. As nested insertions from most orders of TEs are known[45-48] (LTR into helitron, helitron into LTR; TIR into LTR, LTR into TIR), we retain TE copies entirely nested within another copy, but remove insertions that overlap boundaries of other copies. When copies overlap, we retain first LTR retrotransposons, next TIR, next SINE and LINE, and finally helitrons. This removal order was chosen to favor TE orders with stronger structural signatures.

Homology Search: After a TE inserts into a position in the genome, it is subject to subsequent mutations. Because features will erode over time, making identification difficult, these changes can complicate its ascertainment by structural methods. To identify these waning TE-derived sequences, we used RepeatMasker (http://www.repeatmasker.org) to mask the B73 RefGen_v4 pseudomolecules with a repeat library consisting of structurally defined TEs. These consist of the filtered TE set described above, but with LTR retrotransposon families containing greater than 10 copies additionally downsampled to reduce computational runtime. This is necessary due to the existence of large families with tens of thousands of nearly identical copies. For these LTR retrotransposon families, we algorithmically selected exemplar elements, based on the length distribution of the TE family. Briefly, we used a Dirichlet Process Prior to identify the most likely number of normal distributions needed to generate the observed length distribution, and identified cluster membership for each element in the family. Then, we selected the copy with a length closest to the mean of each inferred normal distribution. These copies were used as exemplars in the homology search.

Comparison of transposable element annotations in v3 and v4: To compare our annotation approach with existing TE annotations generated based on homology to the

MTEC repeat library (www.maizetedb.org), we annotated the AGPv3 assembly using the structural methods applied to AGPv4. We then assessed the overlap between the available RepeatMasker annotation of AGPv3 and this new annotation. This analysis revealed that only 0.6% (11,017 of 1,695,362) of LTR retrotransposons in RepeatMasker AGPv3 annotation are full-length and contain TSDs. Such striking underrepresentation is anticipated when homology-based methods are used to identify diverse TEs[49]. In addition to the improved quality of the annotation, the AGPv4 genome allows more complete reconstruction of the entire sequence of each TE. For example, we recovered 68% more Ty1/Copia and Ty3/Gypsy LTR retrotransposons with evidence of all proteins required for retrotransposition (42,929 in AGPv4 vs. 25,412 in AGPv3); in AGPv3, many of these internal domains were represented by gaps between contigs.

Diversification of maize LTR retrotransposons: To investigate the evolutionary dynamics of retrotransposition in maize since divergence from sorghum, we applied our annotation approach for LTR retrotransposons to the *Sorghum bicolor* genome (Sorbi1). Sequences matching HMM models of RT_crm.hmm (Ty3/Gypsy) and RT_sire.hmm (Ty1/Copia) were extracted from each non-nested LTR TE they matched. As the estimated divergence time between maize and sorghum (12 Mya) predicts greater divergence than the 80% identity used to define families, generated a consensus sequence for each family using emboss cons[50] to track differences between species. We aligned these family consensuses with MAFFT mafft[51] and built a maximum likelihood phylogenetic tree with fasttree2[52]. We then collapsed sister tips on the tree if they arose from the same species, and summed the number of copies belonging to each of these species-specific lineages. Hence, monophyletic lineages of TEs, with respect to the genome they were ascertained from, are shown in Figure 2.

Data Availability: Scripts, parameters, and intermediate files of each TE superfamily are available at https://github.com/mcstitzer/agpv4_te_annotation/tree/master/ncbi_pseudomolecule

Reference

1 Berlin, K. *et al.* Assembling large genomes with single-molecule sequencing and locality-sensitive hashing. *Nature biotechnology* **33**, 623-630, doi:10.1038/nbt.3238 (2015).

2 Chin, C. S. *et al.* Nonhybrid, finished microbial genome assemblies from long-read SMRT sequencing data. *Nat Methods* **10**, 563-569, doi:10.1038/nmeth.2474 (2013).

3 Nguyen, J. V. *Genomic mapping: a statistical and algorithmic analysis of the optical mapping system*. (University of Southern California, 2010).

4 Anantharaman, T. & Mishra, B. in *Algorithms in Bioinformatics, First International Workshop, WABI.* 27-40.

5 Myers, E. W. *et al.* A whole-genome assembly of Drosophila. *Science* **287**, 2196-2204 (2000).

6 Sommer, D. D., Delcher, A. L., Salzberg, S. L. & Pop, M. Minimus: a fast, lightweight genome assembler. *BMC bioinformatics* **8**, 64, doi:10.1186/1471-2105-8-64 (2007).

7 Ganal, M. W. *et al.* A large maize (Zea mays L.) SNP genotyping array: development and germplasm genotyping, and genetic mapping to compare with the B73 reference genome. *PLoS One* **6**, e28334, doi:10.1371/journal.pone.0028334 (2011).

8 Gent, J. I., Wang, K., Jiang, J. & Dawe, R. K. Stable Patterns of CENH3 Occupancy Through Maize Lineages Containing Genetically Similar Centromeres. *Genetics* **200**, 1105-1116, doi:10.1534/genetics.115.177360 (2015).

9 Heinz, S. *et al.* Simple combinations of lineage-determining transcription factors prime cis-regulatory elements required for macrophage and B cell identities. *Molecular cell* **38**, 576-589, doi:10.1016/j.molcel.2010.05.004 (2010).

10 Li, H. *et al.* The Sequence Alignment/Map format and SAMtools. *Bioinformatics* **25**, 2078-2079, doi:10.1093/bioinformatics/btp352 (2009).

11 Haberer, G. *et al.* Structure and architecture of the maize genome. *Plant Physiol* **139**, 1612-1624, doi:10.1104/pp.105.068718 (2005).

12 Fouquet, R. *et al.* Maize rough endosperm3 encodes an RNA splicing factor required for endosperm cell differentiation and has a nonautonomous effect on embryo development. *The Plant cell* **23**, 4280-4297, doi:10.1105/tpc.111.092163 (2011).

13 Cantarel, B. L. *et al.* MAKER: an easy-to-use annotation pipeline designed for emerging model organism genomes. *Genome research* **18**, 188-196, doi:10.1101/gr.6743907 (2008).

14 Monaco, M. K. *et al.* Gramene 2013: comparative plant genomics resources. *Nucleic Acids Res* **42**, D1193-1199, doi:10.1093/nar/gkt1110 (2014).

15 Wang, B. *et al.* Unveiling the complexity of the maize transcriptome by single-molecule long-read sequencing. *Nature communications* **7**, 11708, doi:10.1038/ncomms11708 (2016).

16 Soderlund, C. *et al.* Sequencing, mapping, and analysis of 27,455 maize full-length cDNAs. *PLoS Genet* **5**, e1000740, doi:10.1371/journal.pgen.1000740 (2009).
17 Law, M. *et al.* Automated update, revision, and quality control of the maize genome annotations using MAKER-P improves the B73 RefGen_v3 gene models and identifies new genes. *Plant Physiol* **167**, 25-39, doi:10.1104/pp.114.245027 (2015).
18 Martin, J. A. *et al.* A near complete snapshot of the Zea mays seedling transcriptome revealed from ultra-deep sequencing. *Scientific reports* **4**, 4519, doi:10.1038/srep04519 (2014).
19 Keller, O., Kollmar, M., Stanke, M. & Waack, S. A novel hybrid gene prediction method employing protein multiple sequence alignments. *Bioinformatics* **27**, 757-763, doi:10.1093/bioinformatics/btr010 (2011).
20 Schnable, P. S. *et al.* The B73 maize genome: complexity, diversity, and dynamics. *Science* **326**, 1112-1115, doi:10.1126/science.1178534 (2009).
21 Vilella, A. J. *et al.* EnsemblCompara GeneTrees: Complete, duplication-aware phylogenetic trees in vertebrates. *Genome research* **19**, 327-335, doi:10.1101/gr.073585.107 (2009).
22 Haas, B. J., Delcher, A. L., Wortman, J. R. & Salzberg, S. L. DAGchainer: a tool for mining segmental genome duplications and synteny. *Bioinformatics* **20**, 3643-3646, doi:10.1093/bioinformatics/bth397 (2004).
23 Youens-Clark, K. *et al.* Gramene database in 2010: updates and extensions. *Nucleic Acids Res* **39**, D1085-1094, doi:10.1093/nar/gkq1148 (2011).
24 Lowe, T. M. & Eddy, S. R. tRNAscan-SE: a program for improved detection of transfer RNA genes in genomic sequence. *Nucleic Acids Res* **25**, 955-964 (1997).
25 Campbell, M. S. *et al.* MAKER-P: a tool kit for the rapid creation, management, and quality control of plant genome annotations. *Plant Physiol* **164**, 513-524, doi:10.1104/pp.113.230144 (2014).
26 Kurtz, S. *et al.* Versatile and open software for comparing large genomes. *Genome biology* **5**, R12, doi:10.1186/gb-2004-5-2-r12 (2004).
27 Istrail, S. *et al.* Whole-genome shotgun assembly and comparison of human genome assemblies. *Proc Natl Acad Sci U S A* **101**, 1916-1921, doi:10.1073/pnas.0307971100 (2004).
28 Zhao, H. *et al.* CrossMap: a versatile tool for coordinate conversion between genome assemblies. *Bioinformatics* **30**, 1006-1007, doi:10.1093/bioinformatics/btt730 (2014).
29 Kumari, S. & Ware, D. Genome-wide computational prediction and analysis of core promoter elements across plant monocots and dicots. *PLoS One* **8**, e79011, doi:10.1371/journal.pone.0079011 (2013).
30 Smith, A. D., Sumazin, P., Xuan, Z. & Zhang, M. Q. DNA motifs in human and mouse proximal promoters predict tissue-specific expression. *Proc Natl Acad Sci U S A* **103**, 6275-6280, doi:10.1073/pnas.0508169103 (2006).
31 Ellinghaus, D., Kurtz, S. & Willhoeft, U. LTRharvest, an efficient and flexible software for de novo detection of LTR retrotransposons. *BMC bioinformatics* **9**, 18, doi:10.1186/1471-2105-9-18 (2008).

32 Steinbiss, S., Willhoeft, U., Gremme, G. & Kurtz, S. Fine-grained annotation and classification of de novo predicted LTR retrotransposons. *Nucleic Acids Res* **37**, 7002-7013, doi:10.1093/nar/gkp759 (2009).

33 Lerat, E., Burlet, N., Biemont, C. & Vieira, C. Comparative analysis of transposable elements in the melanogaster subgroup sequenced genomes. *Gene* **473**, 100-109, doi:10.1016/j.gene.2010.11.009 (2011).

34 Llorens, C. *et al.* The Gypsy Database (GyDB) of mobile genetic elements: release 2.0. *Nucleic Acids Res* **39**, D70-74, doi:10.1093/nar/gkq1061 (2011).

35 Wenke, T. *et al.* Targeted identification of short interspersed nuclear element families shows their widespread existence and extreme heterogeneity in plant genomes. *The Plant cell* **23**, 3117-3128, doi:10.1105/tpc.111.088682 (2011).

36 Han, Y., Burnette, J. M., 3rd & Wessler, S. R. TARGeT: a web-based pipeline for retrieving and characterizing gene and transposable element families from genomic sequences. *Nucleic Acids Res* **37**, e78, doi:10.1093/nar/gkp295 (2009).

37 Han, Y. & Wessler, S. R. MITE-Hunter: a program for discovering miniature inverted-repeat transposable elements from genomic sequences. *Nucleic Acids Res* **38**, e199, doi:10.1093/nar/gkq862 (2010).

38 Ye, C., Ji, G. & Liang, C. detectMITE: A novel approach to detect miniature inverted repeat transposable elements in genomes. *Scientific reports* **6**, 19688, doi:10.1038/srep19688 (2016).

39 Xiong, W., He, L., Lai, J., Dooner, H. K. & Du, C. HelitronScanner uncovers a large overlooked cache of Helitron transposons in many plant genomes. *Proc Natl Acad Sci U S A* **111**, 10263-10268, doi:10.1073/pnas.1410068111 (2014).

40 Wicker, T. *et al.* A unified classification system for eukaryotic transposable elements. *Nature reviews. Genetics* **8**, 973-982, doi:10.1038/nrg2165 (2007).

41 Baucom, R. S. *et al.* Exceptional diversity, non-random distribution, and rapid evolution of retroelements in the B73 maize genome. *PLoS Genet* **5**, e1000732 (2009).

42 Sweredoski, M., DeRose-Wilson, L. & Gaut, B. S. A comparative computational analysis of nonautonomous helitron elements between maize and rice. *BMC genomics* **9**, 467, doi:10.1186/1471-2164-9-467 (2008).

43 Yang, L. & Bennetzen, J. L. Distribution, diversity, evolution, and survival of Helitrons in the maize genome. *Proc Natl Acad Sci U S A* **106**, 19922-19927, doi:10.1073/pnas.0908008106 (2009).

44 Miele, V., Penel, S. & Duret, L. Ultra-fast sequence clustering from similarity networks with SiLiX. *BMC bioinformatics* **12**, 116, doi:10.1186/1471-2105-12-116 (2011).

45 Gupta, S., Gallavotti, A., Stryker, G. A., Schmidt, R. J. & Lal, S. K. A novel class of Helitron-related transposable elements in maize contain portions of multiple pseudogenes. *Plant molecular biology* **57**, 115-127 (2005).

46 Morgante, M. *et al.* Gene duplication and exon shuffling by helitron-like transposons generate intraspecies diversity in maize. *Nat Genet* **37**, 997-1002 (2005).

47 Jameson, N. *et al.* Helitron mediated amplification of cytochrome P450 monooxygenase gene in maize. *Plant molecular biology* **67**, 295-304 (2008).

48 Jiang, N. & Wessler, S. R. Insertion preference of maize and rice miniature inverted repeat transposable elements as revealed by the analysis of nested elements. *The Plant cell* **13**, 2553-2564 (2001).

49 Platt, R. N., Blanco-Berdugo, L. & Ray, D. A. Accurate transposable element annotation is vital when analyzing new genome assemblies. *Genome Biology and Evolution*, evw009 (2016).

50 Rice, P., Longden, I. & Bleasby, A. EMBOSS: the European molecular biology open software suite. *Trends in genetics* **16**, 276-277 (2000).

51 Katoh, K. & Standley, D. M. MAFFT multiple sequence alignment software version 7: improvements in performance and usability. *Molecular biology and evolution* **30**, 772-780 (2013).

52 Price, M. N., Dehal, P. S. & Arkin, A. P. FastTree 2–approximately maximum-likelihood trees for large alignments. *PLoS One* **5**, e9490 (2010).
