# Supplementary figures and images for: Improved maize reference genome with single-molecule technologies
Source: Nature. 2017 Jun 12;546(7659):524–7. doi: 10.1038/nature22971 (PMC7052699; doi:10.1038/nature22971)

## Slide 1
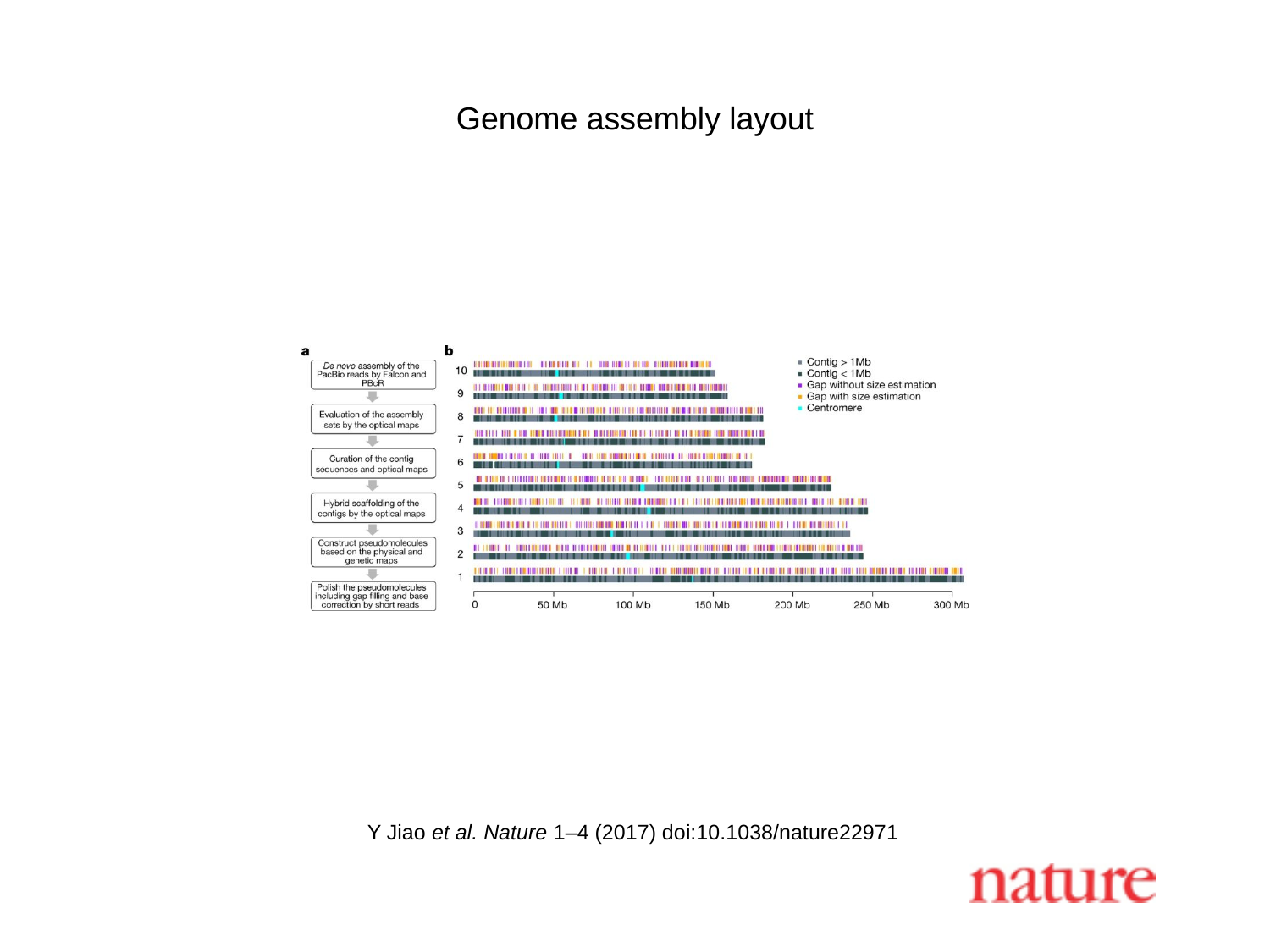

# Genome assembly layout
Y Jiao et al. Nature 1–4 (2017) doi:10.1038/nature22971

Supplement: Supplementary file 2 — PowerPoint slide for Fig. 1 [file 41586_2017_BFnature22971_MOESM2_ESM.ppt]

## Slide 1
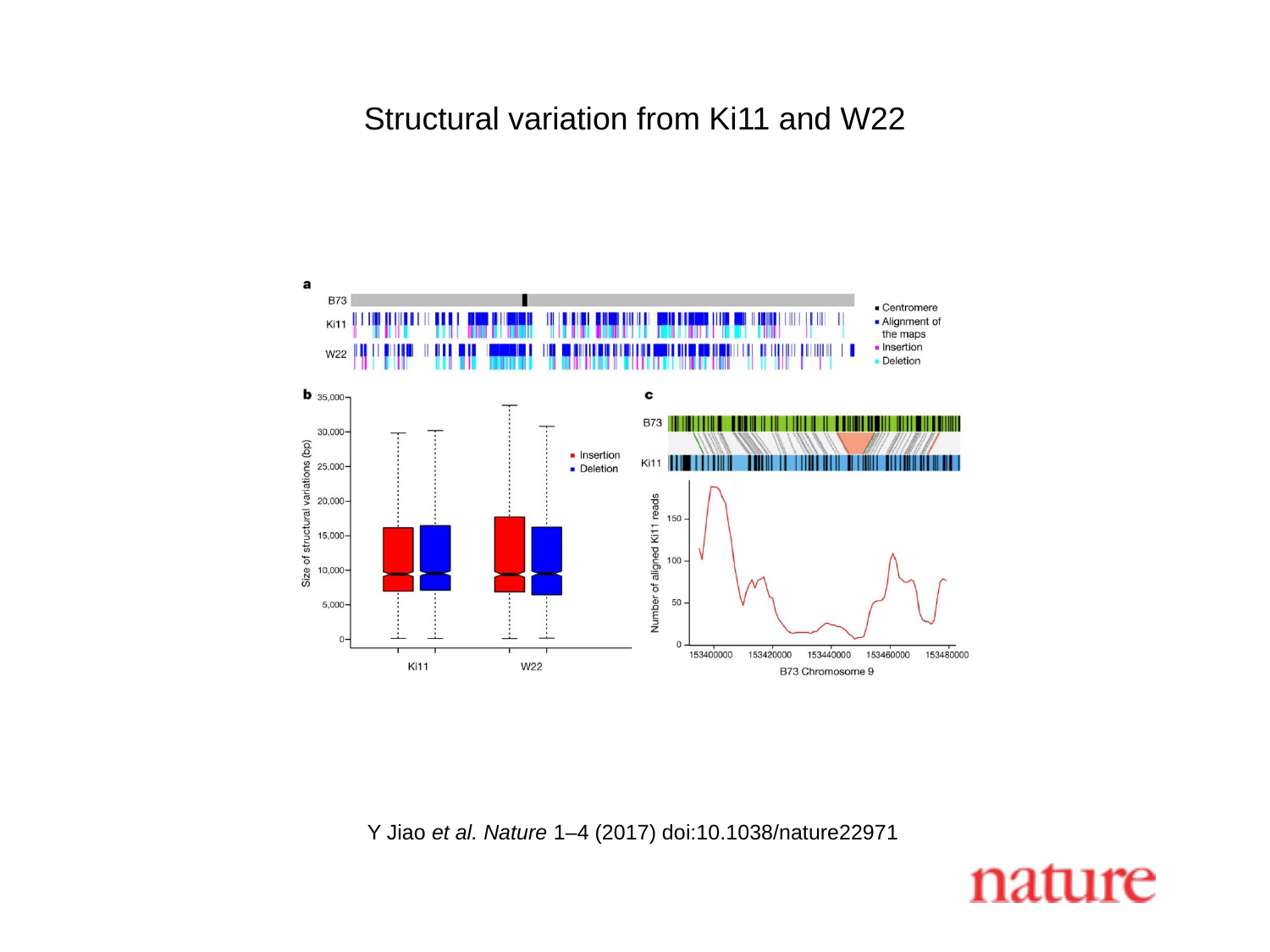

# Structural variation from Ki11 and W22
Y Jiao et al. Nature 1–4 (2017) doi:10.1038/nature22971

Supplement: Supplementary file 4 — PowerPoint slide for Fig. 3 [file 41586_2017_BFnature22971_MOESM4_ESM.ppt]
