# Supplementary material for: Improved maize reference genome with single-molecule technologies
Source: Nature. 2017 Jun 12;546(7659):524–7. doi: 10.1038/nature22971 (PMC7052699; doi:10.1038/nature22971)
Supplement: Supplementary file 3 — PowerPoint slide for Fig. 2 [file 41586_2017_BFnature22971_MOESM3_ESM.ppt]

## Slide 1
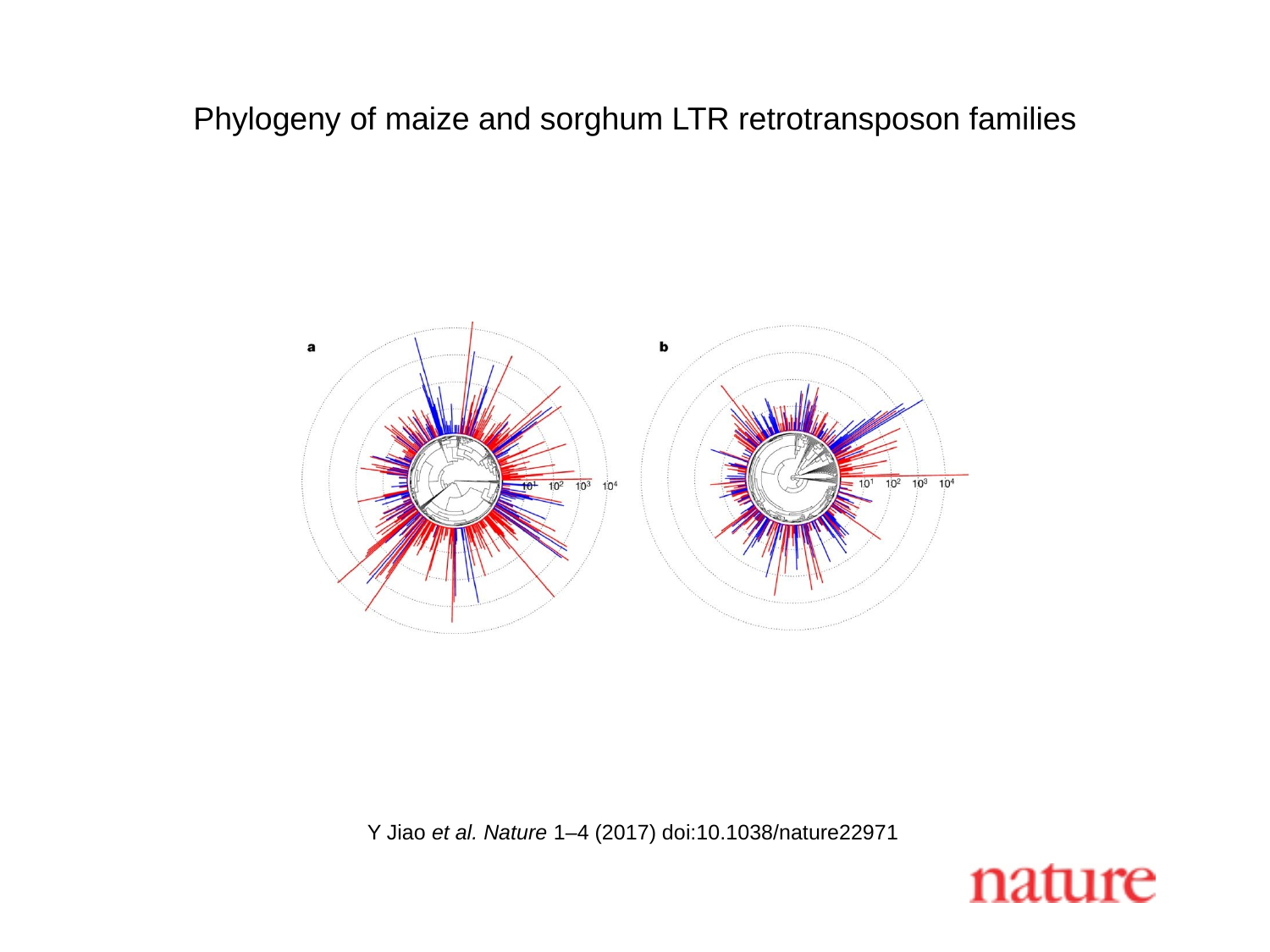

# Phylogeny of maize and sorghum LTR retrotransposon families
Y Jiao et al. Nature 1–4 (2017) doi:10.1038/nature22971
